# Supplementary material for: Artificial Intelligence Based Algorithms for Prostate Cancer Classification and Detection on Magnetic Resonance Imaging: A Narrative Review
Source: Diagnostics (Basel). 2021 May 26;11(6):959. doi: 10.3390/diagnostics11060959 (PMC8229869; doi:10.3390/diagnostics11060959)
Supplement: Supplementary file 1 [file diagnostics-11-00959-s001.zip › diagnostics-1231170-supplementary.pdf]

Artificial Intelligence Based Algorithms for Prostate Cancer Classification and Detection on Magnetic Resonance Imaging: A Narrative Review

Jasper J. Twilt [1,*], Kicky G. van Leeuwen [1] , Henkjan J. Huisman [1] , Jurgen J. Fütterer [1] and Maarten de Rooij [1]

Supplementary Materials

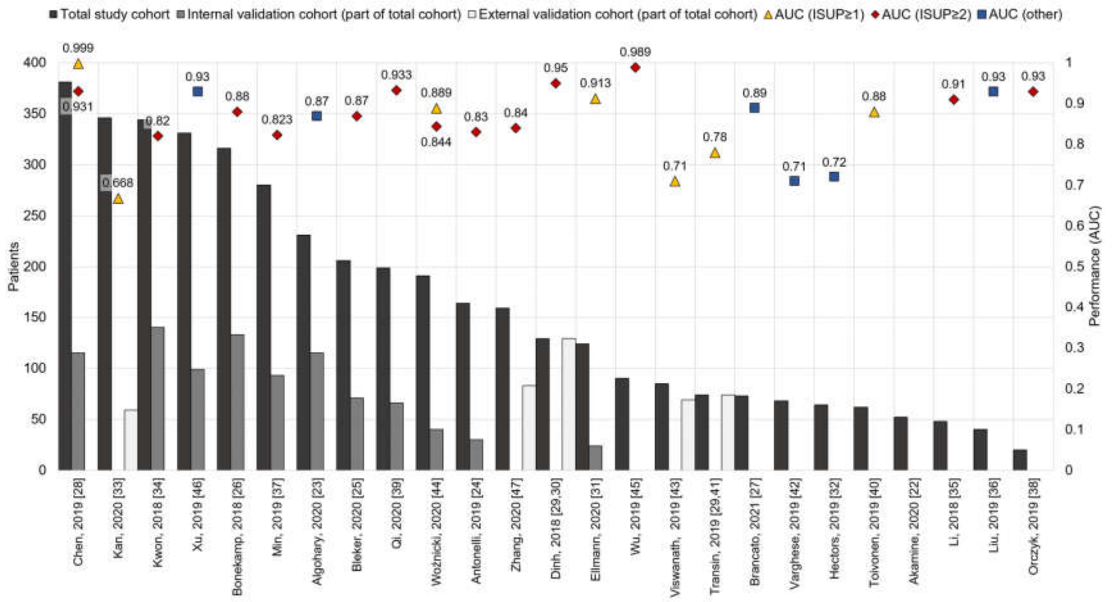

Figure S1. Observed heterogeneity in study cohort, validation cohort and validation approach for two-class lesion classification algorithms utilizing machine learning. Studies are ranked according to total cohort size and shown with corresponding validation cohort, validation approach and reported area under the ROC curve (AUC) for various ISUP thresholds, when available. Cohort size ranged from 20 to 381 patients (median=129). Distinction between internal validation (i.e. utilizing the same dataset for training and validation) and external validation (i.e. utilizing unseen data as compared to training data), is visualized. If no validation cohort is visualized, cross-validation methods were applied. Studies with total study cohort and validation cohort equal, represented validation of an existing algorithm in a new cohort.

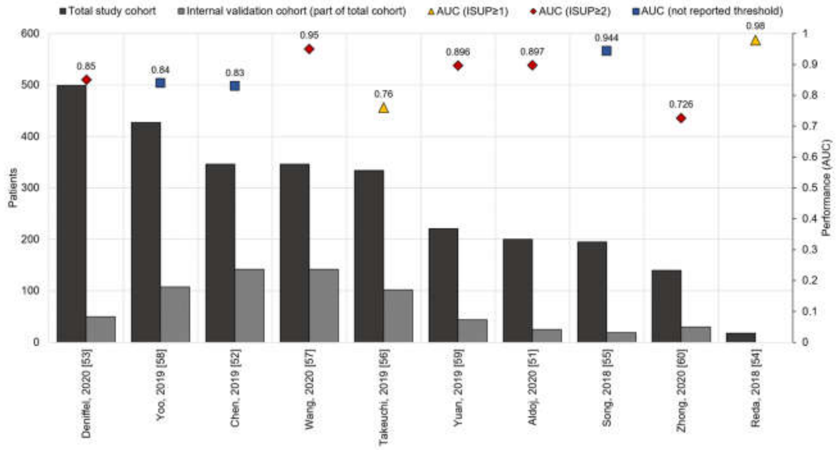

Figure S2. Observed heterogeneity in study cohort, validation cohort and validation approach for two-class lesion classification algorithms utilizing deep learning. Studies are ranked according to total cohort size and shown with corresponding validation cohort, validation approach and reported area under the ROC curve (AUC) for various ISUP thresholds, when available. Cohort size ranged from 18 to 499 patients (median = 278). For all studies, internal validation was performed (i.e. utilizing the same dataset for training and validation).

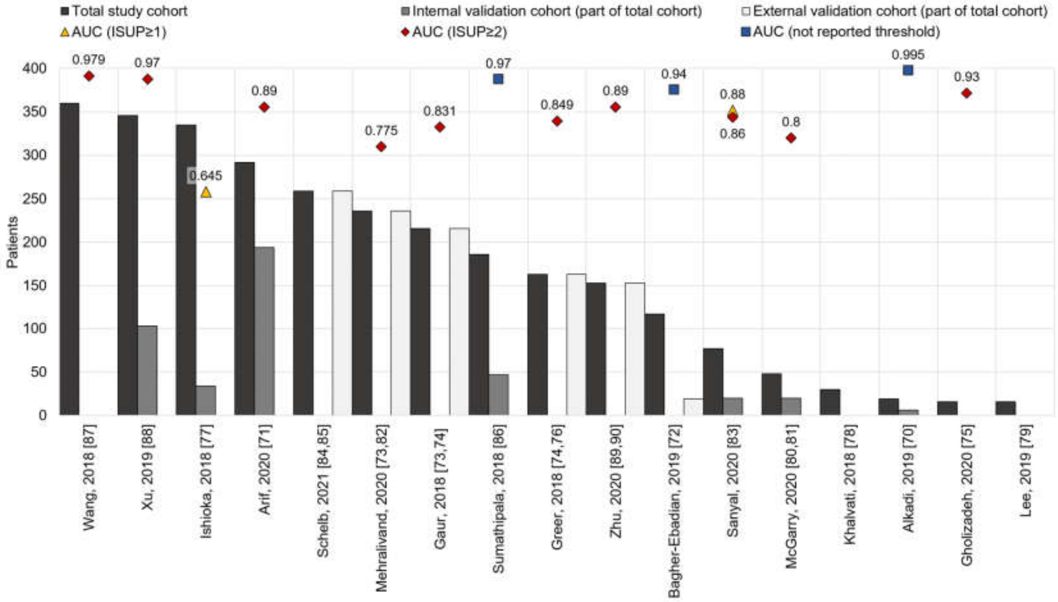

Figure S3. Observed heterogeneity in study cohort, validation cohort and validation approach for two-class lesion detection algorithms. Studies are ranked according to total cohort size and shown with corresponding validation cohort, validation approach and reported area under the ROC curve (AUC) for various ISUP thresholds, when available. Cohort sizes ranged from 16 to 360 patients (median=163). Distinction between internal validation (i.e. utilizing the same dataset for training and validation) and external validation (i.e. utilizing unseen data as compared to training data), is visualized. If no validation cohort is visualized, cross-validation methods were applied. Studies with total study cohort and validation cohort equal, represented validation of an existing algorithm in a new cohort.
